# Anti-quorum sensing and antibiofilm activity of coumarin derivatives against *Pseudomonas aeruginosa* PAO1: Insights from *in vitro* and *in silico* studies

**DOI:** 10.22038/IJBMS.2023.69016.15047

**Published:** 2023-04

**Authors:** Amineh Sadat Tajani, Zeinab Amiri Tehranizadeh, Arianoosh Pourmohammad, Armin Pourmohammad, Milad Iranshahi, Faegheh Farhadi, Vahid Soheili, Bibi Sedigheh Fazly Bazzaz

**Affiliations:** 1 Department of Pharmaceutical Control, School of Pharmacy, Mashhad University of Medical Sciences, Mashhad, Iran; 2 Department of Medicinal Chemistry, School of Pharmacy, Mashhad University of Medical Sciences, Mashhad, Iran; 3 Department of Pharmacognosy, School of Pharmacy, Mashhad University of Medical Sciences, Mashhad, Iran; 4 Biotechnology Research Center, Pharmaceutical Technology Institute, Mashhad University of Medical Sciences, Mashhad, Iran

**Keywords:** Biofilm, Coumarin, Microbial resistance, *Pseudomonas aeruginosa*, Quorum sensing

## Abstract

**Objective(s):**

Biofilm-associated infections are challenging to manage or treat since the biofilm matrix is impenetrable to most antibiotics. Therefore, the best approach to deal with biofilm infections is to interrupt the construction during the initial levels. Biofilm formation has been regulated through the quorum sensing (QS) network, making it an attractive target for any antibacterial therapy.

**Materials and Methods:**

Here, some coumarin members, including umbelliprenin, 4-farnesyloxycoumarin, gummosin, samarcandin, farnesifrol A, B, C, and auraptan, have been assessed as QS inhibitors *in silico* and *in vitro.* Their potential inhibitory effects on biofilm formation and virulence factor production of *Pseudomonas aeruginosa *PAO1 were evaluated.

**Results:**

First, the interaction of these compounds was investigated against one of the major transcriptional regulator proteins, PqsR, using molecular docking and structural analysis methodology. After that, *in vitro *evaluations indicated that 4-farnesyloxycoumarin and farnesifrol B showed considerable reduction in biofilm formation (62% and 56%, respectively), virulence factor production, and synergistic effects with tobramycin. Moreover, 4-farnesyloxycoumarin significantly (99.5%) reduced *PqsR* gene expression.

**Conclusion:**

The biofilm formation test, virulence factors production assays, gene expression analysis, and molecular dynamic simulations data demonstrated that coumarin derivatives are a potential anti-QS family through PqsR inhibition.

## Introduction

Over the last few decades, emerging of antibiotic resistance has become a universal problem for public health and health professionals (1). In most cases, the foremost underlying cause of this resistance is the antibiotic’s mode of action, i.e., killing bacteria or inhibiting bacterial growth. Since selective pressure was increased in bacterial populations through antibiotics, the vulnerable bacteria were omitted, and the percentage of resistant bacteria continued to grow (2). 

*Pseudomonas aeruginosa* is from the gammaproteo-bacteria class, belonging to the *Pseudomonadaceae *family, which contributes to the development of broad antibiotic resistance. *P.* *aeruginosa* is an opportunistic, Gram-negative, aerobic, rod-shaped, and mobile microorganism characterized by a single polar flagellum. Due to its extensive metabolic diversity, this microorganism can grow in various environments and food sources. *P. aeruginosa* is a common cause of acute and chronic infections from the community and the hospital, including burn wounds and urinary tract infections, bacteremia, pneumonia, and various systemic infections, especially in immunocompromised patients (3). This pathogen produces a series of virulence factors and biofilm to overcome the defense of host cells regulated through a developed cell-to-cell signaling system named quorum sensing (QS). QS is an intercellular communication employed by many bacteria to detect population density through receiving and sending “autoinducer” signals that coordinate collective behaviors (4). *P. aeruginosa* has three major QS systems: *las*, *rhl*, and *pqs *(5). According to studies, all three quorum-sensing systems play essential roles in the biofilm life cycle (3, 6). It can form biofilm in less than 4 hr, which plays a significant role in antibiotic resistance. Each cascade comprises a transcriptional regulator protein named LasR, RhlR, and PqsR, which are activated with their corresponding signaling molecule. While Acyl-homoserine lactones (3- oxo-C12-AHL and C4-HSL) are autoinducers for LasR and RhlR systems; 3,4-dihydroxy-2- heptylquinoline (PQS), its precursor 2-heptyl-4-hydroxyquinoline (HHQ), and 2-nonyl-4-hydroxyquinoline (NHQ) are natural ligands of PqsR. HHQ, PQS, and NHQ not only promote their own production but also develop colony biofilm formation in the primary stages of *Pseudomonas* infection and inflammation (5, 7). Accordingly, it is expected that the PqsR inhibitors significantly reduce biofilm formation. QS in *P. aeruginosa* also regulates production of virulence factors such as protease, rhamnolipids, alkaline protease, lectins, superoxidase dismutases, exotoxin A, hydrogen cyanide, and pyocyanin (3).

The biofilm matrix commonly contains extracellular polymeric substances (EPS) (composed of polysaccharides (like alginate), proteins, etc.) and extracellular DNA (eDNA) (8). The EPS layer of biofilm, which is behind the biofilm connection to solid surfaces and its maturation, supports bacteria to survive in unfavorable environments (9).

Moreover, this layer is the main reason for bacterial resistance to antimicrobials. It accumulates the antibacterial agents up to 25% of its weight, thereby slowing antibacterial transportation and accelerating their destruction with the help of exoenzymes (9). Cells in biofilm undergo metabolism changes, genetic alterations, transgenic gene transfer, and persister cell production depending on environmental conditions. On the other hand, biocides usually kill metabolically active bacteria, and dormant growth cells are less sensitive to antimicrobials (10).

QS inhibition has been suggested as a promising antivirulence strategy to disarm bacteria instead of killing them, and a wide range of potential QS inhibitors have been studied (11-14)

**Figure 1:**
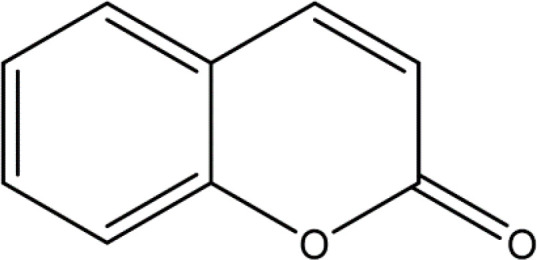
Core structure of the coumarin family

Coumarins are a family of natural origin that are biosynthesized by plant tissues and produced in response to infection (phytoalexins). The core structure of coumarin consists of a benzene ring attached to an alpha-pyrone ring and can be classified as benzopyrones (Figure 1). 

**Figure 2:**
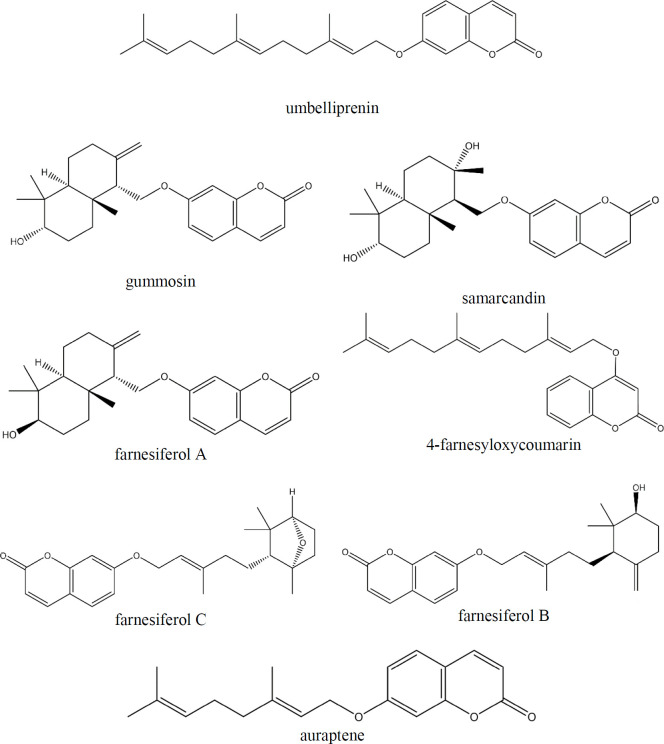
Chemical structure of coumarin derivatives participates in this study

Coumarins have many attractive characteristics, including low molecular weight, low toxicity, high bioavailability, simple structure, and good solubility in most organic solvents. Moreover, various types of substitutions in the basic structure can influence their biological activities. Different biological activities have been listed for these molecules, including anticancer, anti-inflammation, antimicrobial, anti-oxidants, pain-killing, anti-arrhythmic, antihypertensive, and anticoagulation (15-17). The effects of coumarins as QS inhibitors (QSI) and antibiofilm agents have reported the potential impact of these natural compounds on suppressing QS manifestations (18-20). In the present study, some coumarins members, including, umbelliprenin, 4-farnesyloxycoumarin, gummosin, samarcandin, farnesiferol A, B, C, and auraptene, have been assessed as QSI, and their potential inhibitory effects on biofilm formation and virulence factors production were evaluated (Figure 2).

## Materials and Methods

***Chemicals and materials***

All coumarin derivatives were extracted according to a previous study (21). Standard *P. aeruginosa *PAO1 (Nottingham wild-type strain) was used in experiments. All antimicrobial assessments were conducted in Mueller-Hinton agar (MHA) and Mueller-Hinton broth (MHB) medium from HiMedia (India). Tobramycin was ordered from Sigma-Aldrich (USA). Other chemicals, including 2,3,5-Triphenyl tetrazolium chloride (TTC), chloroform, HCl, and skimmed milk, were purchased from Merck (Germany).

***Molecular docking***

Molecular docking (MD) calculations were performed to compare the interaction energies of the selected coumarin derivatives and NHQ by MOE software version 2019 (22). Initially, the three-dimensional structure of the PqsR protein from the Protein Data Bank was procured (PDB-ID: 4JVD). Then, the ligand and water molecules were all eliminated from the crystal 3D structure of PqsR’s PDB file. Moreover, the ligand and protein were optimized and prepared for the docking process. The protein structure’s breaks or defects were also modified using the MOE-Quick preparation tool. Eventually, the ligands were docked in the PqsR protein through the MOE-Dock tool, and the rescoring functions, London dG, and GBVI/WSA dG were employed for pose scoring (23, 24).

***Molecular dynamic simulation***

The best scoring results of MD were introduced into GROMACS 2018, as well as the CHARMM36 force field, and the protein-ligand complexes’ stability and accuracy were determined (25, 26). The systems were placed within a cubic unit cell of a SPC/E water model with an adequate amount of neutralizing ions. Moreover, the whole system was equilibrated at a 300 K temperature and 1 bar pressure, and the energy was minimized under the Steepest Descent algorithm. Afterward, NVT and NPT were applied as the equilibration steps before the MD simulation to equilibrate the entire system (27). Eventually, all protein-ligand complexes were subjected to 100 nano-seconds for molecular dynamic simulation. The ligands’ capacity in PqsR inhibition was considered through RMSD and Lennard-Jones’ potential analyzing processes.

***Minimum inhibitory concentration (MIC) determination of coumarin derivatives and tobramycin***

A 2 mg/ml stock solution was made from all coumarin derivatives and tobramycin in a glucose-enriched MHB medium supplemented with DMSO as a co-solvent. Concentrations of 1, 0.8, 0.6, 0.4, and 0.2 mg/ml of each coumarin were prepared from stock solutions. Moreover, various concentrations of tobramycin were prepared serially. 

For each concentration of the selected coumarins, tobramycin, positive, and negative control, three wells were considered in a 96-well plate and were filled with 180 µl of prepared solutions. Then, 20 µl of 10^6^ CFU/ml bacterial suspension was inoculated to each well (except for negative control), and the plate was incubated for 24 hr at 37 °C. After that, 20 µl of the aqueous solution of TTC (5 mg/ml) was added to the content of each well. Following two more hours of incubation at 37 °C, the lowest concentration, whose red color was not observed, was reported as the MIC (13).

***Biofilm inhibition***

Each selected coumarin (4-farnesyloxycoumarin, gummosin, and farnesifrol A, B) was made either 100 and 200 μg/ml or just 200 μg/ml in sterile glucose-enriched MHB in the presence of 1% DMSO. Then, 180 µl of the prepared concentration was inoculated with 20 µl of bacterial suspension (10^6^ CFU/ml) in a sterile flat-bottom 96-well plate and was incubated for 24 hr at 37 °C. In the next step, the content of each well was gently drained and washed three times with sterile normal saline. After drying, the wells were stained with crystal violet solution (0.1% w/v) for 15 min and washed again till the extra dye was removed. At last, the remaining dye trapped in biofilm matrices was dissolved in 96% ethanol, and after 15 min, the absorbance was read at 590 nm by an ELISA reader (Synergy H4, USA) (14).

***Virulence factor production***

*Preparation of cell-free supernatant*

For each selected coumarin compound and positive control, 30 ml of glucose-enriched MHB medium was inoculated with 250 µl of bacterial suspension containing 10^10^ CFU/ml *P. aeruginosa* PAO1 (no inoculation for the negative control). The flasks were incubated in a shaker incubator at 37 °C and 200 rpm. After 24 hr, the content of each flask was centrifuged at 10,000 rpm for 10 min, and the cell-free supernatant was discarded. After that, the bacterial pellet was resuspended in 10 ml of fresh glucose-enriched MHB in the presence of 200 μg/ml of each selected substance. The flasks were covered entirely with aluminum foil and incubated again at 37 °C and 200 rpm in a shaker. After 24 hr, the contents of each flask were centrifuged at 10,000 rpm for 10 min. The cell-free supernatant was separated from the bacterial pellet and filtered (syringe filter 0.22 μm). Then it was stored at 4 °C for further study (28).

*Assay of pyocyanin*

In order to extract pyocyanin from the cell-free supernatant, 5 ml was transferred to sterile tubes, and 2 ml of chloroform was added to each. The blue chloroform phase, which is located at the bottom of the tube, was removed. This process was repeated two more times. Then, 1 ml hydrochloric acid (0.2 N) was added to the chloroform phase (three times), and the resultant pink layer was separated. Extraction steps were also performed for both positive and negative controls. The optical density of each prepared sample was measured at 388 nm through an ELISA reader (Synergy H4, USA) (13).

*Assay of pyoverdine*

The volume of 200 µl from the cell-free supernatant was transferred into a black microtiter 96-well plate, and its fluorescence emission was measured by an ELISA reader (λ_ex_ = 400 nm and λ_em_ = 455 nm). The results were compared with the control groups (positive and negative) (28).

The percentage of inhibition for pyocyanin and pyoverdine was calculated according to the following equation:

$\mathrm{\% Inhibition for pyocyanin or pyoverdine production}=\frac{\mathrm{Positive control}-\mathrm{sample absorption}}{\mathrm{Positive Control}}\times 100$

*Assay of total protease*

 One ml skimmed milk (1.5%) was added to 5 ml cell-free supernatant and incubated at 37 °C to analyze the secreted protease. After 2 hr, 200 µl of each sample was inoculated in wells (three wells for each selected material, positive control, and negative control), and the optical density was measured by an ELISA reader at a wavelength of 500 nm (13). The absorbance values were expressed as % inhibition for protease activity through the following equation:

$\mathrm{\% Inhibition for protease activity}=1-\frac{\mathrm{negative control}-\mathrm{sample absorption}}{\mathrm{Positive Control}-\mathrm{Negative control}}\times 100$

***Coumarins potentiation effect with tobramycin against sessile bacteria***

In order to evaluate the potentiation effect of the selected coumarins, bacteria were cultured in a 96-well plate according to the biofilm formation test. After 24 hr, the culture medium was replaced with a fresh medium and incubated again at 37 °C for 12 hr. Finally, the content of each well was drained and refilled with 200 μl of MHB containing selected coumarins as well as tobramycin at MIC (2 μg/ml). After 12 more hours of incubation, the medium of the wells was changed with fresh MHB, 20 μl of TTC aqueous solution (5 mg/ml) was added, and the plate was incubated again. After 3 hr, the red color intensity of each well was measured at 450 nm (13).

***Evaluating the effect of coumarin derivatives on the expression of PqsR genes***

*Extraction of total RNA and synthesis of cDNA*

The impact of 4-farensiloxycoumarin on the relative expression of PqsR protein genes was determined using real-time polymerase chain reaction (RT-PCR). *P. aeruginosa* PAO1 was grown in liquid glucose-enriched MHB at 37 °C and 200 rpm for 24 hr with and without 4-farensiloxycoumarin. Bacterial cultures were centrifuged, and total RNA was extracted from the cell pellets with a Column RNA Extraction Kit (Dena Zist Asia, Iran) through the manufacturer’s description. The purity and quality of the extracted RNA were assessed by Nanodrop (Thermo Fisher Scientific, Finland). The extracted RNA was reverse transcribed into cDNA through the Easy cDNA Synthesis Kit (Parstous, Iran).

*RT-PCR of the PqsR gene*

The RT-PCR was carried out via the Applied Biosystems, the StepOne™ (48-well) Real-Time PCR System (reaction was set up using Super SYBR Green qPCR MasterMix 2X (Yekta Tajhiz Azma, Iran)) with the following primers: 5’-AACATGTTCCTCCAGGTCAT-3’ as forward primer and 5’-GTTGAGATTGAAGGCGATGT-3’ as reverse primer. The gene expression level was relatively normalized to the expression of the housekeeping gene. The expression of *PqsR* genes in *P. aeruginosa* cultivated with coumarins was compared with the expression of control cultures without coumarin treatment.

***Statistical analysis***

The results of coumarin’s effect on *P. aeruginosa* pathogenicities were precisely analyzed using SPSS software (version 16) using one-way ANOVA (Tukey or Tamhane tests), where *P*<0.05 was taken as statistically significant. 

## Results

***Molecular docking***

**Figure 3:**
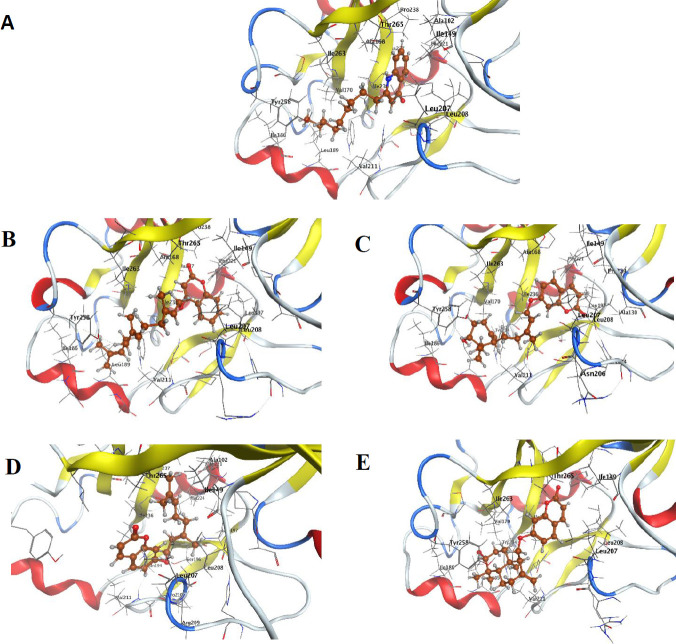
PqsR active site in the presence of the natural ligand NHQ (A) and the coumarin derivatives, 4- farensiloxycoumarin (B), farensyferol B (C), auraptene (D), and gummosin (E)

Four coumarin derivatives (gummosin, auraptene, farensyferol B, and farensiloxycoumarin) were docked in the active site of the PqsR protein and compared with NHQ (natural ligand). The best docking results and similarity in the interaction with the pocket belong to farensiloxycoumarin (7.77 kcal/mol) compared with NHQ (Figure 3).

***Molecular dynamic simulation***

The simulation results indicated that all the coumarin-PqsR complexes were stable, and the difference in position in the active site caused their altered effects. 

***Root mean square deviation (RMSD)***

**Figure 4:**
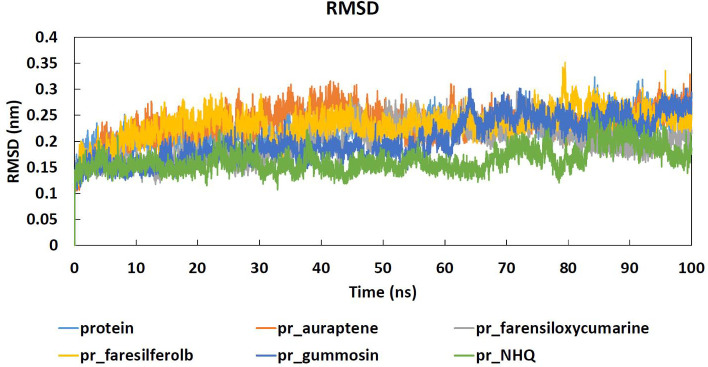
RMSDas diagram of all interactions between the PqsR active site and coumarin derivatives

The diagram shows that the coumarin-PqsR complexes’ stabilities were 0.1–0.25 nm, similar to protein alone during the 100 ns simulations (Figure 4).

***Lennard-Jones potential***

The best LJ potential energy belongs to 4-farensiloxycoumarin (-182.8 ± 2.6) compared with NHQ (-126.377 ± 1.5). The other coumarin derivatives’ LJ potential energies were in the range of -140 to -165.

***MIC determination of coumarin derivatives and tobramycin***

The results indicated that none of the tested compounds at concentrations of 1, 0.8, 0.6, 0.4, and 0.2 mg/ml could inhibit *P. aeruginosa* PAO1 growth, and the red color due to the reduction of TTC to formazone was observed in all wells. The MIC of tobramycin was determined as 2 µg/ml.

***Effect of coumarin derivatives on biofilm inhibition***

**Figure 5:**
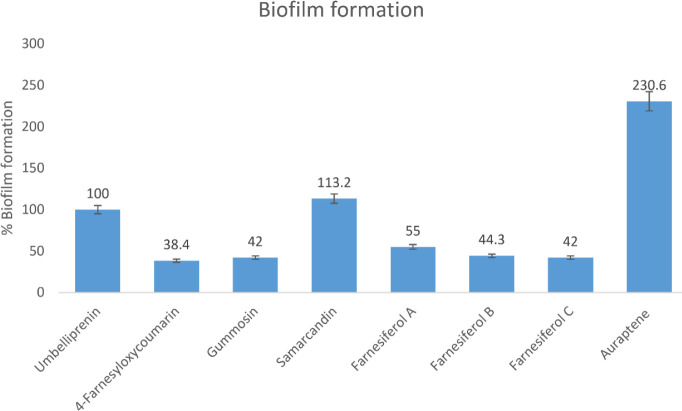
Amount of biofilm formed by *Pseudomonas aeruginosa* in the presence of coumarin derivatives

The effects of sub-MIC concentration of coumarin derivatives on biofilm formation were evaluated through the crystal violet method, and the results were analyzed through SPSS software. The processed data indicated that 4-farnesyloxycoumarin, gummosin, farnesiferol A, B, and C significantly (*P*<0.05) decreased biofilm construction by 62%, 58%, 45%, 56%, and 58%, respectively, compared with the positive control (Figure 5). Therefore, these compounds were selected for the subsequent assays. Samarcandin and auraptene have significantly increased biofilm formation, and umbelliprenin showed no significant effect. Thus, umbelliprenin, samarcandin, and auraptene were excluded from further studies due to their undesirable impacts.

***Effect of coumarin derivatives on virulence factors production***

**Figure 6.**
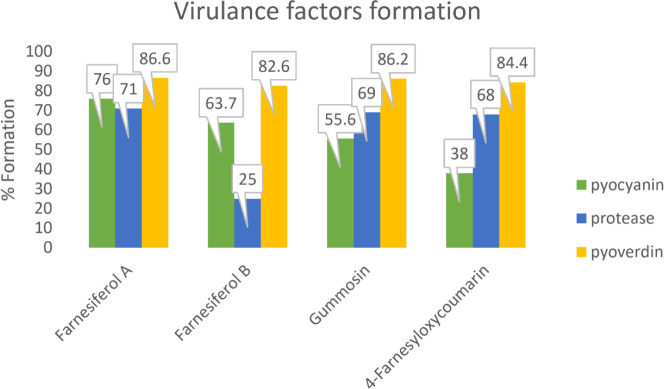
Amount of the virulence factors produced in *Pseudomonas aeruginosa *culture in the presence of coumarin derivatives

Analyzing the mean absorbance data showed that pyocyanin production was significantly reduced by about 62%, 44%, 24%, and 36.3% in the presence of 4-farnesyloxycoumarin, gummosin, farnesiferol A, and B, respectively (*P*<0.05) (Figure 6). In addition, based on Figure 6, pyoverdine concentration was also significantly decreased through coumarin treatment. 4-Farnesyloxycoumarin reduced pyoverdine by about 16%, gummosin by 14%, followed by 13%, and 17% for farnesiferol A and B, respectively. As indicated in Figure 6, the protease production was significantly (*P*<0.05) suppressed by 200 µg/ml of 4-farnesyloxycoumarin, gummosin, and farnesiferol A and B. In fact, 32% inhibition of protease activity for 4-farnesyloxycoumarin, 31% for gummosin, 29% for farnesiferol A, and 75% for farnesiferol B was observed.

***Effect of coumarin derivatives on increasing the efficiency of tobramycin against bacteria in biofilm***

**Figure 7:**
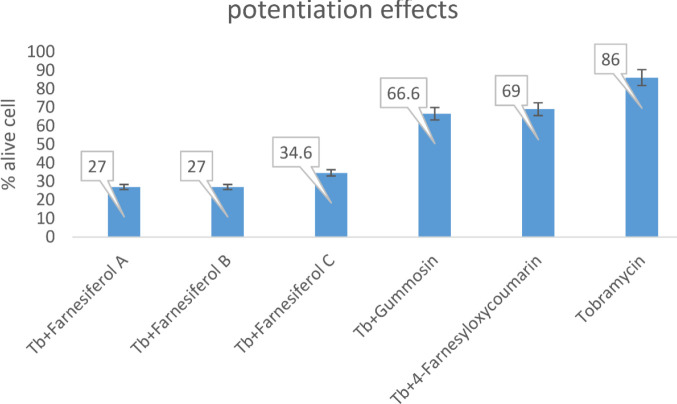
Potentiation of tobramycin (Tb) activity by coumarin derivatives against *Pseudomonas aeruginosa*

The 200 μg/ml of coumarin derivatives along with a MIC of tobramycin, increase the penetration and the efficacy of tobramycin. The results indicated that the mean absorbance for tobramycin with coumarin derivatives compared with the positive control and tobramycin alone significantly decreased (*P*<0.05). Therefore, all the coumarin derivatives decreased the viable cells compared with tobramycin and positive control (Figure 7).

***Evaluating the effect of coumarin derivatives on the expression of PqsR genes***

Based on the obtained results, 4-farnesyloxycoumarin significantly reduced the expression ratio present in the *PqsR* gene to 99.562% compared with the control group with no treatment.

## Discussion

The potential anti-QS and antibiofilm activities of coumarins were first assessed virtually and reported by Zeng *et al.* in 2008 (28). Although they did not explore these effects experimentally, they indicated that esculetin and esculin could inhibit the formation of *P. aeruginosa* biofilm and promote the proteolysis of TeaR (a signal receptor protein in *Escherichia coli*) (29). After that, many researchers evaluated coumarin, its derivatives potential as QSI, and its manifestations. In particular, coumarin displayed inhibitory effects on biofilm formation, production of virulence factors (pyocyanin and pyoverdin), and motility in *P. aeruginosa* PA14 (19). Moreover, it turned out that several essential genes involved in the *Pseudomonas* QS system would be down-regulated in coumarin-treated culture (30). Following that, D’Almeida *et al.* showed that coumarin and hydroxylated coumarin derivatives (esculetin and umbelliferone) presented the highest biofilm inhibition; on the other hand, 4-hydroxycorumarin and dihydrocoumarin indicated the lowest effect in this regard. They concluded that the hydroxyl group on the coumarin aromatic ring displays a significant role in inhibiting the biofilm formation of *P. aeruginosa* (31). These recent reports on the antibiofilm and anti-QS effects of coumarin compounds have encouraged us to study the coumarin derivatives, the inhibitory effects of which on the *Pseudomonas* QS network and biofilm formation have previously not been investigated.

The MD approach was brought to predict the interaction between any ligands (natural ligands like NHQ or coumarin derivatives) and PqsR at the atomic level, which also characterize the ligand’s behavior in the binding site elucidating the best inhibitor. Based on the previous studies, NHQ (natural PqsR agonist) is precisely positioned in the PqsR active site in the two sub-pockets A and B (7). The quinolone moiety on NHQ is enclosed in the B pocket by contacts from Ile 236, Leu 207, and Leu 208. The alkyl chain forms a comfortable fit in pocket A by making interaction with Tyr 258, Ile 186, Val 170, Leu 189, and Ile 236. In the coumarin family, the oxocoumarin bicyclic ring is placed in the B pocket, the same as the quinoline in NHQ, except for urapten, wherein the alkyl chain is located. This molecular misrotation was observed despite the docking process repetition, which may be due to the presence of double bonds in the alkyl chain. The greatest conformational relationship and similarity in the interaction with the pocket residues were seen in 4-farensiloxycoumarin compared with NHQ (Figure 3).

Root-mean-square deviation active site (RMSDas) of the PqsR active site was calculated to investigate the conformation and stability conversions of active site residues, as well as the connection with ligands. In interpreting the RMSD results, the small value describes a highly complex stability. The result indicated that all the coumarin-PqsR complexes indicated acceptable stabilities. After that, the Lennard-Jones (LJ) potential describes the potential energy of a pair of non-bonding molecules or atoms based on their separation distance. The LJ negative values indicate superior stability with higher attraction. In this test, the most negative result was related to 4-farensiloxycoumarin. So, in all molecular simulation tests, 4-farensiloxycoumarin indicated the best results compared with the natural ligand, NHQ.

In the next level, the MIC of the selected compounds was determined. MIC is defined as the minimum concentration of the substance that inhibits bacterial growth. Therefore, the minimum concentration of each tested coumarin, without color changing to red, was selected as the MIC. Since the red color was observed in all wells, it was concluded that the compounds have no significant antibacterial properties, and none of them can be used as possible antibiotics. As there were no antimicrobial effects, these compounds could be considered QS inhibitors, but further assessments should be performed. In this study, the selected coumarin derivatives dramatically decreased biofilm formation (45–62%), whereas, in another study, bergamottin and dihydroxybergamottin (two other coumarin derivatives) indicated only 18.1% and 27.3% biofilm inhibition in *P. aeruginosa*, respectively (32). The best reduction result belonged to 4-farensiloxycoumarin (62%)( Figure 5).

After that, the inhibitory effect of the selected coumarins on QS-controlled virulence factors, including pyocyanin, pyoverdin, and protease, was investigated. Since pyocyanin is essential for biofilm maturation and infection, any reduction in its amount can significantly reduce *Pseudomonas* pathogenicity (33). Pyocyanin is a blue, water-soluble, and non-fluorescent pigment with a phenazine structure that plays a vital role in biofilm formation, and *P. aeruginosa* solely produces it (34)*.* Compared with all coumarin derivatives, 4-farnesyloxycoumarin was more effective in lowering pyocyanin production (Figure 6). 

The yellow-green fluorescent siderophore, entitled pyoverdine, enabled Fe(III) ion acquisition from the bacterial media and also played as a signaling molecule in the *Pseudomonas* QS system (35). All coumarin derivatives have shown almost the same efficiency in reducing the virulence factor pyoverdine compared with the positive control. Farnesiferol A, B, and 4-farensiloxycoumarin exhibited the best results in lowering pyoverdine production (17 and 16%, respectively) (Figure 6).

In evaluating the effect of the coumarin derivatives on total protease production compared with the positive control, the higher the mean absorbance, the better the inhibitory effect of the coumarins. In fact, the more produced protease by bacteria in the samples results in extra-degradation of skimmed milk proteins and a further reduction in optical density. Protease production was significantly suppressed by all the tested coumarins derivatives, especially farnesiferol B (75%) and 4-farensiloxycoumarin (32%) (Figure 6).

The potentiation effects of tobramycin with coumarins were also evaluated. Biofilm production by *P. aeruginosa* dramatically reduces the tobramycin effect on bacteria. The higher the mean absorbance of TTC, the more bacteria were viable in the sample (28) and the less effective the coumarins were. Statistical analysis of the results showed that the mean absorbance of the positive control and tobramycin alone are almost similar, meaning that the biofilm was resistant to antibiotic infiltration. However, coumarin addition to the bacterial media, significantly decreased the number of viable cells by increasing the antibiotic penetration (Figure 7).

Finally, the gene expression assay was also applied to evaluate the effects of coumarin derivatives on the expression of the *PqsR* gene of *P. aeruginosa*. 4-Farnesyloxycoumarin exhibited antagonistic interaction. This antagonistic effect of 4-farnesyloxycoumarin plays a crucial role since the formation of a complex between PqsR and its natural ligands (AQs) triggers the positive regulation of several genes, including the production of virulence factors like pyocyanin, elastase, hydrogen cyanide, lectins, and the biosynthesis of *pqsABCDE* operon. Moreover, iron usage regulation in *P. aeruginosa *is under PqsR activity. On the other hand, this transcriptional regulator also auto-regulates the synthesis of its ligands. Therefore, any blocking is capable of reducing both the pathogenicity and resistance of this recalcitrant bacterium (5, 6).

## Conclusion

New antimicrobial strategies are being developed, considering the emergence of multi-drug-resistant pathogens. For centuries, natural-based compounds have been a great choice due to the deep connection between human health and natural medicine. Overall, our results presented new data about the anti-QS activity of coumarin derivatives against *P. aeruginosa*. The biofilm formation test, virulence factor production assays, gene expression analysis, and molecular dynamic simulations data demonstrated that coumarin derivatives are a potential anti-QS family through PqsR inhibition. Moreover, along with other results, the cotreatment of *P. aeruginosa* with tobramycin and coumarins has provided enough evidence to nominate these compounds for antimicrobial clinical trials. Among these derivatives, 4-farnesyloxycoumarin, gummosin, and farnesifrol B and C indicated a considerable reduction in biofilm formation, virulence factor production, and synergistic effects with tobramycin. Additionally, 4-farnesyloxycoumarin indicated high potency in reducing the *PqsR* gene expression. 

## Authors’ Contributions

AS T conceived the study, performed experimental work, and wrote the original draft. ZAT supervised molecular modeling calculations, critical revision, and final approval of the manuscript. AP and AP performed experimental work. MI and FF formulated overarching research goals and methodology and provided coumarin derivatives. VS helped with funding acquisition, formulation of overarching research goals and methods, supervision, and critical revision. BSFB provided supervision, conceptualization, and critical revision. All authors have read and confirmed the final version of the manuscript.

## Data Availability

The datasets have been generated and are available from the corresponding authors on reasonable request.

## Conflicts of Interest

The authors declare that no conflict of interest exists.
